# The Complement System Is Essential for the Phagocytosis of Mesenchymal Stromal Cells by Monocytes

**DOI:** 10.3389/fimmu.2019.02249

**Published:** 2019-09-20

**Authors:** Caroline Gavin, Stephan Meinke, Nina Heldring, Kathleen Anne Heck, Adnane Achour, Ellen Iacobaeus, Petter Höglund, Katarina Le Blanc, Nadir Kadri

**Affiliations:** ^1^Department of Laboratory Medicine, Karolinska Institutet, Stockholm, Sweden; ^2^Department of Medicine, Huddinge, Karolinska Institutet, Stockholm, Sweden; ^3^Science for Life Laboratory, Division of Infectious Diseases, Department of Medicine Solna, Karolinska Institutet, Karolinska University Hospital, Stockholm, Sweden; ^4^Division of Neurology, Department of Clinical Neuroscience, Karolinska Institutet, Stockholm, Sweden; ^5^Clinical Immunology and Transfusion Medicine, Karolinska University Hospital, Stockholm, Sweden; ^6^Center of Hematology, Karolinska University Hospital, Stockholm, Sweden

**Keywords:** MSC, phagocytosis, monocytes, complement, fate, plasma, live

## Abstract

Mesenchymal stromal cell (MSC) therapy is a promising tool in the treatment of chronic inflammatory diseases. This has been ascribed to the capacity of MSC to release a large variety of immune-modulatory factors. However, all aspects of the mode of therapeutic MSC action in different diseases remain unresolved, mainly because most of the infused MSC are undetectable in the circulation within hours after infusion. The aim of this study was to elucidate the fate of MSC after contact with plasma. We found that upon contact with blood, complement proteins including C3b/iC3b are deposited on MSC. Importantly, we also found that complement bound to MSC enhanced their phagocytosis by classical and intermediate monocytes via a mechanism that involves C3 but not C5. Thus, we describe for the first time a mechanism which might explain, at least partly, why MSC are not found in the blood circulation after infusion. Our results indicate that MSC immune-modulatory effects could be mediated by monocytes that have phagocytosed them.

## Introduction

Mesenchymal stromal cells (MSC) have emerged as a possible new treatment for several chronic inflammatory diseases including diabetes, graft versus host disease, and multiple sclerosis (1). Their immune-modulatory function has mainly been ascribed to paracrine mechanisms associated with secretion of immunoregulatory mediators including cytokines and growth factors which modulate inflammatory response and balance immune profiles (2). The soluble immune secretomes include prostaglandin E2 (PGE-2), indoleamine 2,3-dioxygenase (IDO), or nitric oxide (NO)(3). In numerous clinical trials, MSC have been infused to the circulation (4) but the infused cells have been difficult to detect in the blood already at short time points after infusion (5). Furthermore, tracing studies of injected MSC have revealed that only few MSC were detectable at the site of injury or inflammation despite encouraging clinical outcomes (6–8). Hence, the actual modes of action of intravenous infusion of MSC in several diseases remain unresolved.

Previous studies have shown that MSC have a very short half-life (9) and that their infusion leads to an instant blood mediated inflammatory reaction (10). Indeed, hypotheses that MSC may be trapped in the lungs where they would interact with local macrophages are gaining in popularity (9, 11, 12). Moreover, Galleu et al. have demonstrated that infused MSC are subject to perforin-induced apoptosis through recipient cytotoxic cells, which favor their phagocytosis by monocytes (13). Also, complement activation by MSC plays a role in immunosuppression of peripheral blood cells via a mechanism that involves CD11b^+^ cells (14). On the other hand, another study also suggested that MSC may get injured after contact with blood compounds due to the complement system (15). Thus, further studies are needed to understand the interactions of MSC with different components of the immune system, in order to shed light on their fate after infusion and their mechanisms of action.

The complement system, which comprises more than 30 proteins, plays an important role in innate immunity during inflammatory responses against foreign agents (16). It can be activated through three different pathways; the classical, the lectin and the alternative pathway. The classical pathway which uses the circulating C1q molecule is mainly activated by antibodies bound to the surface of a target cell. The lectin pathway uses mannose-binding lectins that bind carbohydrate molecules at the surface of various pathogens. The alternative pathway is constitutively active at a low level in normal serum via spontaneous hydrolysis of C3. Each of these three pathways leads to the generation of labile C3 convertases, which cleave C3 into C3a and C3b that can thereafter participate in forming distinct complexes. Ultimately, the complement cascade results in activation of C5 that initiates the formation of the C5b-7 complex that finally forms the membrane attack complex (MAC), resulting in cell lysis (17). Complement activation is regulated by soluble and cell surface-bound complement inhibitors, which limit uncontrolled complement activation. These complement regulators, including CD46 and CD55, prevent C3b which binds to the host surface, from either forming C3 convertases or from initiating decay of the complexes (18). Other complement regulators such as CD59 prevent MAC assembly and pore formation in the cell membrane (19).

Receptors for complement components have been previously described in various cell types including monocytes. The complement receptor 3 (CR3), comprising CD11b, and CD18, is expressed by all monocytes and critical for facilitating phagocytosis of complement-opsonized cells or pathogens (20). In addition, an increased percentage of suppressive cells including M2 monocytes was found *in vivo* after MSC infusion (13, 21–23). Thus, we here hypothesized that MSC interact with complement components in plasma, which might facilitate their phagocytosis by monocytes, explaining their disappearance directly after infusion. We here demonstrate that live complement-opsonised MSC are phagocytosed by classical CD14^+^CD16^−^ and intermediate CD14^+^CD16^−^ monocytes via a mechanism that involves C3 but not C5.

## Materials and Methods

### MSC Donors, Isolation, and Expansion

The study was approved by the Stockholm regional ethics committee. All patients provided written consent (ethical permit number: DNR 2016/338-32-4). Human bone marrow (BM) derived MSC were isolated from 12 healthy volunteer donors as described previously (24). Briefly, under local anesthesia, 30–50 mL aspirate was obtained from posterior iliac crest bone marrow (BM). MSC were isolated from the BM-mononuclear cell (MNC) fraction by Percoll density gradient centrifugation. Cells were washed and expanded in Dulbecco's modified Eagle's medium (DMEM) low-glucose complete medium, supplemented with 10% heat inactivated fetal calf serum and antibiotic-antimycotic (A/A; Gibco, Grand Island, NY), and plated at a density of 1.7 × 10^5^ cells per cm^2^. Cells were prepared for harvest, washed with phosphate-buffered saline (PBS) and detached with 0.05% Trypsin-EDTA (Gibco, Grand Island, NY) for maximum 10 min at 37°C, thereafter replated at a density of 3,400–4,000 cells per cm^2^ and detached at a minimum confluence of 70%. Cells were either replated or cryopreserved in 10% DMSO/DMEM complete medium until further use, in liquid nitrogen. The guidelines of the International Society for Cellular Therapy were applied to analyse the MSC prior to use in research. For *in vitro* assays, MSC from passage 2–4 were thawed in DMEM complete medium on the day of experiments. Cultures were performed under sterile conditions in humidified atmosphere at 37°C in 5% CO_2_. Co-culture experiments were carried out in 96-well-plates (Costar Ultra-low Cluster, Corning) in Roswell Park Memorial Institute 1640 (RPMI) GlutaMAX® (Gibco, Grand Island, NY) complete medium, supplemented with 10% heat-inactivated pooled human blood type AB serum or 10% FCS, penicillin (100 U/mL) and streptomycin (0.1 mg/mL).

### Plasma Preparation

Thrombin inhibitor Lepirudin (Refludan®) was added immediately to fresh peripheral blood samples obtained from healthy volunteers. The samples were centrifuged at 2,000 × g for 10 min at 4°C. The plasma was removed and kept on ice until further use. To focus on the complement system and exclude the coagulation cascade, we used a thrombin inhibitor in both the blood and plasma experiments. Heat inactivated (HI) plasma (30 min at 60°C) or K_3_EDTA (final concentration of 10 mM, pH 7.3, Alfa Aesar) were used as negative controls. C3 inhibitor (10 μM, Compstatin, CP-20 a generous gift from Professor John D. Lambris, Professor of Research Medicine in the Department of Pathology & Laboratory Medicine at the University of Pennsylvania, Philadelphia, PA, USA) or C5 inhibitor (250 μg/mL, Eculizimab, Soliris, Alexion Pharmaceuticals) were used in order to inhibit the binding of complement factor C3 or C5 to the cell surface.

### Blood-Chamber and Blood Isolation Procedure

The blood chamber technique has been previously described (25). Briefly, thrombin inhibitor Lepirudin (final concentration 50 μg/mL [50 mg in 1 mL NaCl]) (Refludan®) was added immediately to fresh peripheral blood obtained from healthy donors, and collected in pre-heparinized tubes. As a negative control K_3_EDTA (pH 7.4) was added at a final concentration of 10 mM. Blood was added into pre-heparinized chambers, where MSC were added and incubated on a rotator at 37°C at different time points. The experiment was stopped by adding K_3_EDTA (pH 7.4). In selected experiments one fraction of MSC was exposed to 10 μg/mL complement inhibitors (all from Biolegend) for 30 min at 4°C. The effect of fresh blood on MSC was assessed for viability and C3b/iC3b binding [revealed using anti-C3c FITC which binds to C3b and iC3b fragments on MSC (14)] using FlowSight system (Merckmillipore). Lysis buffer (BD Pharm Lyse®, BD Biosciences) was used to remove red blood cells before antibody staining.

### MSC Expansion and Differentiation

MSC were thawed and seeded in DMEM culture for 1 week. MSC were trypsinised, washed and cultured in DMEM (Gibco) containing 50% plasma ± 10 mM K_3_EDTA, HI plasma or DMEM complete medium alone for 1, 3, or 24 h. Cells were centrifuged, washed and plated in fresh DMEM complete medium, and thereafter examined for adhesion to plastic and expansion for 6 days. Expanded MSC were used for subsequent *in vitro* experiments. Retained differentiation capacity of MSC was assessed using media and instruction protocols from either adipogenic (Stempro, Invitrogen) or osteogenic (Miltenyi Biotech, GmbH). Adipocyte and osteocyte differentiations were evaluated by Oil Red O (Sigma-Aldrich) and alkaline phosphatase (Sigma Fast, BCIP/NBT), respectively. Presence of lipid vacuoles or calcium deposits was analyzed under a wide field optical microscope.

### T Cell Stimulation and Suppression Assay

PBMCs were freshly isolated from buffy coats using density gradient centrifugation on Ficoll-Isopaque (Lymphoprep® Axis-Shield, Norway), according to the manufacturer's protocol. Human CD3^+^ T cells were isolated by negative selection (Miltenyi Biotec; Human Pan T Cell Isolation Kit) according to the manufacturer's instructions. CD3^+^ T cells (purity >95%) were then stained with carboxyfluorescein succinimidyl ester (CFSE) (Invitrogen) and activated using anti-CD3/CD28 microbeads (Miltenyi Biotec) for 5 days. Using flow cytometry (BD Fortessa LSR-II), proliferation of T cells was assessed in the presence or absence of MSC. All antibodies used for T cell staining are presented in Table 1. Depending on conditions, MSC were treated for 1 h with plasma or heat-inactivated plasma prior to co-culture with T cells. Data were analyzed using FlowJo software (Ashland, OH).

**Table 1:** Antibodies used in the current study.

**Target**	**Fluorochrome**	**Clone**	**Dilution**	**Company**
C3c	FITC		1:100	Dako
CD3	APC-Cy7	OKT3	1:100	BD Biosciences
CD3	PerCP-Cy5.5	OKT3	1:100	BD Biosciences
CD4	PE-Cy5	OKT4	1:200	BD Biosciences
CDS	Alexa488	RPA-T8	1:400	BD Biosciences
CD11b	FITC	M1/70	1:100	Biolegend
CD14	PerCp-Cy5.5	HCD14	1:200	Biolegend
CD16	PE-CF594	3G8	1:400	BD Biosciences
CD32	PE	FUN-2	1:100	Biolegend
CD46	FITC	MEM-258	1:100	Biolegend
CD55	PE-Cy7	JS11	1:100	Biolegend
CD59	PE	p282(H19)L	1:200	Biolegend
CD64	PE-Cy7	10.1	1:200	Biolegend
CD73	APC-Cy7	AD2	1:100	Biolegend
CD73	FITC	AD2	1:100	Biolegend
CD73	APC	AD2	1:100	BD Biosciences
IDO	Alexa Fluor 488		1:100	RD systems
IL-6	PE-CF594	MQ2-1	1:100	BD Biosciences
LIVE/DEA D™ Fixable	V525		1:1,000	Invitrogen
7AAD	PerCp-Cy5.5		1:100	BD Pharmingen

### Cell Surface Staining

MSC treated with plasma as described above were stained with MSC markers described in Table 1. Briefly, after 20 min of incubation at 4°C with specific antibodies, cells were centrifuged for 5 min at 400x g at 4°C. The supernatant was removed and the cell pellet of each well was taken up in 200 μL PBS. The contents of each well were then acquired using flow cytometry (BD LSRFortessa, BD Biosciences) and data were analyzed using the FlowJo software (Ashland, OH).

### Licensing Assay

MSC production of interleukin (IL)-6 and Indoleamine 2,3-dioxygenase (IDO) in response to licensing by proinflammatory stimuli was assessed after exposure to plasma. MSC were thawed and exposed to 50% plasma ± 10 mM K_3_EDTA or 50% heat inactivated plasma. As negative control, MSC were cultured in DMEM complete medium only for 1 h as described for previous experiments. Cells were washed, replated and thereafter licensed with 10 ng/mL tumor necrosis factor (TNF)-α, and 100 U/mL interferon (IFN)-γ for 72 h. For detection of intracellular IL-6 or IDO, GolgiPlug™ (BD Biosciences) was added 5 h prior to the end of the experiment (according to manufacturer's protocol). Cells were acquired using flow cytometry (BD LSR Fortessa, BD Biosciences) and data were analyzed using the FlowJo software (FlowJo, Ashland, OH).

### Complement Lysis Assay

Freshly thawed MSC were loaded with calcein red-orange acetoxymethyl ester (calcein RO AM) (Molecular Probes) at a concentration of 2.5 μg/mL in PBS, and incubated for 10 min at 37°C. Cells were then centrifuged and resuspended in PBS (control), in 50% plasma or in 50% heat-inactivated plasma as negative control. The blocking antibodies CD46, CD55, or CD59 against complement regulators (Table 1) were added and the experiment was analyzed after 1 h incubation at 37°C. Staining for flow cytometry was performed as described above.

### Phagocytosis Assay

Freshly thawed MSC were stained with 51 nmol/L pHrodo succinimidyl ester (Molecular Probes) in PBS for 10 min at RT, centrifuged at 500 × g for 7 min and resuspended in DMEM. Cells were incubated for 1 h at 37°C with either of the following conditions: control DMEM complete medium, or medium with 50% plasma, 50% HI plasma or 50% plasma with 10 mM K_3_EDTA. MSC under each condition were also divided up in the following fractions: MSC alone, MSC with C3 inhibitor (10 μM, Compstatin) or MSC with C5 inhibitor (250 μg/mL, Soliris) added, respectively. One further fraction was exposed to complete medium with added C3 complement protein (15 mg/mL, Sigma C2910). MSC were washed and resuspended in complete DMEM medium. The cell line THP-1 or freshly isolated PBMCs containing monocytes were used as phagocytes. To increase the phagocytic activity of THP-1 cells, 15 ng/mL phorbol 12-myristate 13-acetate were added for 15 min at 37°C. Further, 10 μg/mL Cytochalasin D (Sigma-Aldrich) was added to negative control cells in order to block phagocytosis for a minimum of 30 min at 37°C. MSC were co-cultured with phagocytic cells at 1:1 ratio and incubated for 2 h at 37°C. Thereafter, cells were centrifuged at 400x g for 5 min and stained on ice for analysis by flow cytometry (BD LSRFortessa, BD Biosciences). Monocytes were identified by forward scatter/side scatter (FSC/SSC) and gating on CD14^+^ cells. Phagocytosis was detected by pHrodo fluorescence. The positive gate was set based on the negative control with Cytochalasin D. Cells were acquired using flow cytometry (BD LSRFortessa, BD Biosciences) and data were analyzed using the FlowJo software (FlowJo, Ashland, OH).

### Statistical Analysis

Statistical analysis using paired *t*-test or one way ANOVA (described in figure legends) were performed using Graph Pad Prism (Graph Pad Prism Software Incl. San Diego, USA). *p* < 0.05 was considered statistically significant.

## Results

### Survival and Function of MSC Upon Contact With Plasma

**Figure 1:**
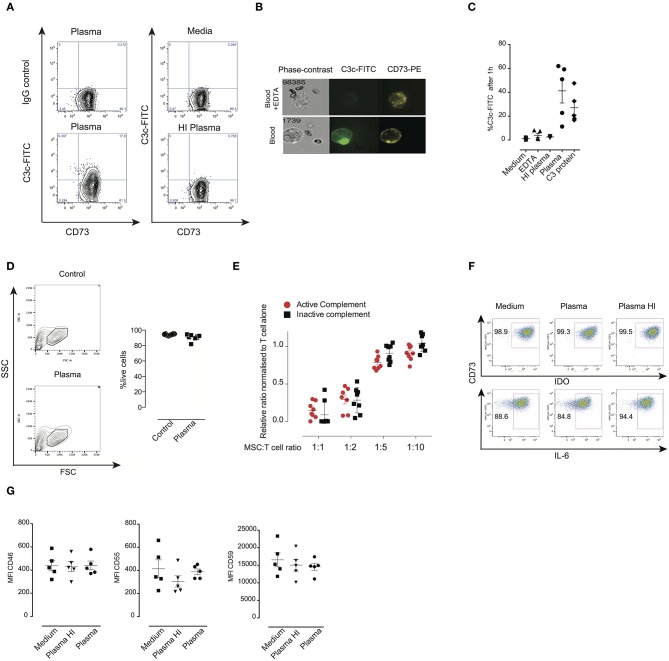
Survival and function of MSC after their contact with plasma. **(A)** Contour plots from flow cytometry analysis of MSC stained with anti-C3c FITC or specific isotope control to detect C3b/iC3b deposition after exposure to plasma, heat inactivated (HI) plasma or untreated MSC. **(B)** FlowSight images of MSC incubated with blood compounds in the presence or absence of 10 mM EDTA for 30 min. MSC were stained with C3c-FITC and CD73-PE. Brightfield image of MSC is shown in the left part of the image. **(C)** Percentage of C3c-FITC binding to MSC after exposure to different conditions (media, EDTA-plasma, HI-plasma, plasma, purified C3 protein) for 1 h. **(D)** Representative contour plot showing FSC and SSC of MSC after their exposure to plasma. Bars represent percentage of live (aqua live dead negative) MSC after 1 h incubation in control vs. plasma from two different experiments. Data shown are representative of two experiments of five different MSC. **(E)** Inhibition of proliferation of 5 days activated T cells (*n* = 3) in the presence of MSC (*n* = 7) at the indicated ratios. Data shown are means and SD of two independent experiments. **(F)** Intracellular IDO or IL-6 expression was measured by flow cytometry in MSC exposed to control media, inactive complement plasma (+ EDTA, 10 mM) or active complement plasma for 1 h, thereafter washed and treated with TNF-α and IFN-γ for 72 h. Data shown are representative from four MSC. **(G)** Expression of complement regulatory proteins CD46, CD55, and CD59 on MSC cultured in complete medium, HI plasma or plasma was analyzed by flow cytometry. Mean fluorescent intensities of MSC from five MSC in two different experiments are displayed with mean and SD.

Due to the fast clearance of MSC from blood circulation after *i.v*. infusion, it remains unclear whether MSC die after contact with plasma. To test this, we incubated MSC with freshly isolated plasma in different time periods stretching from one up to 24 h (Figures 1A–C) and data not shown. It has been recently demonstrated that the anti-human C3c antibody detects C3b/iC3b deposition on the surface of MSC after incubation with serum (14). To check for potential MSC interaction with the complement system, we stained for the presence of the complement components C3b/iC3b on the surface of MSC by using the same antibody (Figures 1A–C). MSC from different healthy donors displayed heterogeneity in C3b/iC3b deposition, ranging from 10 to 60% after 1 h of incubation with plasma (Figure 1C). Heterogeneity of C3b/iC3b deposition was also observed when we used purified C3 protein together with MSC (Figure 1C). Using flow cytometry, we found that MSC incubated with plasma displayed the same shape and granularity with no significant changes in viability compared to control MSC (Figure 1D). This was also observed when MSC were incubated with all blood compounds using the blood chamber technique (25) (Figure 1B and data not shown**)**. It should be noted that the intact coagulation system was inhibited in all experiments by the addition of a thrombin inhibitor. Altogether, these results indicate that MSC survive *in vitro* during the first hours when they interact with plasma.

Moreover, we found that MSC pre-treated with plasma suppressed the proliferation of activated CD3^+^ T cells similarly to MSC pre-treated with heat inactivated (HI) plasma or non-treated MSC, suggesting that the immune-suppressive properties of MSC are not altered after interaction with plasma compounds (Figure 1E). In response to high concentrations of IFNγ, and TNFα, MSC pre-incubated with plasma still produced substantial levels of IL-6 and IDO (Figure 1F). Using the appropriate media for MSC differentiation, we found that MSC pre-incubated with plasma differentiate to adipocytes and chondrocytes similarly to control MSC suggesting that the differentiation capacity of MSC was maintained (Data not shown). Interestingly, expression levels of the complement regulators CD46, CD55, and CD59 were not affected following contact between MSC and plasma (Figure 1G). Altogether, these results indicate that MSC survive and are fully functional, after contact with plasma and that this protection might be due to the expression of complement regulators.

### Effects of Blocking Complement Regulators on MSC Pre-treated With Plasma

**Figure 2:**
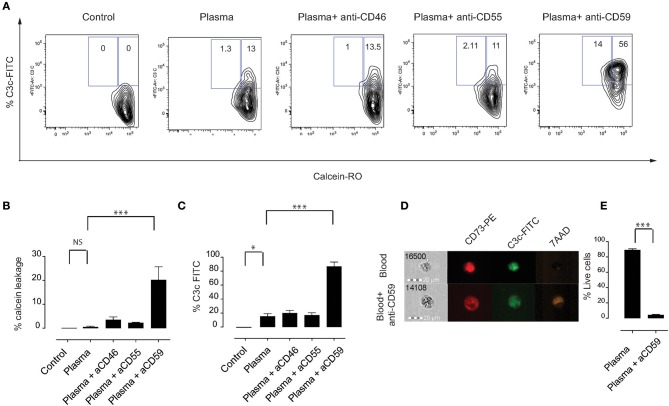
Effects of blocking complement regulators on MSC pre-treated with plasma. **(A)** Representative contour plots of calcein RO-stained MSC exposed to plasma, HI plasma or medium for 1 h in the presence or absence of blocking antibodies against complement inhibitors CD46, CD55, or CD59. **(B)** Percentage of calcein leakage, and **(C)** percentage of C3b/iC3b binding to MSC pre-treated with anti-CD46, anti-CD55, and anti-CD59 blocking antibodies in the presence of active plasma. Data shown are means and SD (*n* = 4). **(D)** FlowSight images showing C3b/iC3b binding and 7-AAD (death marker) on MSC in the presence or absence of anti-CD59 blocking antibody in the blood chamber experiments. **(E)** Percentage of live cells (aqua live dead negative) of MSC pre-treated with plasma in the presence or absence of anti-CD59 blocking antibodies. Data shown are means and SD (*n* = 4). Data are representative of two independent experiments. Statistical significance was determined using paired *t*-test *p = 0.05 and ***p = 0.0001.

We tested whether expression of complement regulators CD46, CD55, and CD59 is important for the survival of MSC following incubation with plasma. MSC were labeled with calcein which is a cytosolic dye that leaks out if the membrane of MSC is damaged by the membrane attack complex (26). MSC were treated with specific blocking antibodies, washed, then incubated later with plasma. Thereafter, MSC were stained for C3b/iC3b deposition on their surface (Figures 2A–C). Addition of an anti-CD59 specific antibody but not anti-CD46 nor anti-CD55 resulted in calcein leakage when MSC were pre-treated with plasma (Figures 2A–C). Importantly, blocking of any complement inhibitor did not induce calcein leakage when MSC were pre-treated with HI plasma or untreated MSC (Figure S1). The shape and granularity of MSC were dramatically changed with an accumulation of cell debris when MSC were pre-treated with plasma and antibodies blocking the complement inhibitor CD59 (Figure S2). Moreover, inhibition of CD59 led to a pronounced increase of C3b/iC3b deposition on the surface of MSC pre-treated with plasma compared to controls (Figures 2A–C). Using FlowSight, we found that blocking of CD59, but not other complement regulators resulted in cell death as shown by 7-AAD staining (Figure 2D) and data not shown. Similarly, using flow cytometry, we found that almost all MSC stained positive for the dead cell marker after pre-treatment with complement inhibitor CD59 and plasma (Figure 2E). CD59 was expressed at a much higher levels compared to CD46 and CD55 on the surface of all analyzed MSC. Thus, the different expression levels of CD46, CD55, and CD59 do not exclude the possibility that both CD46 and CD55 may also be important for protection. Furthermore, although this result is interesting, it should be noted that blocking of CD59 by this specific IgG2a isotype may also induce killing of MSC through the activation of the classical pathway (27). Thus, at this stage, our results which suggest that CD59 may protect MSC from complement lysis should be considered as suggestive, but not conclusive.

### Complement Factors Enhance Phagocytosis of MSC by Monocytes

**Figure 3:**
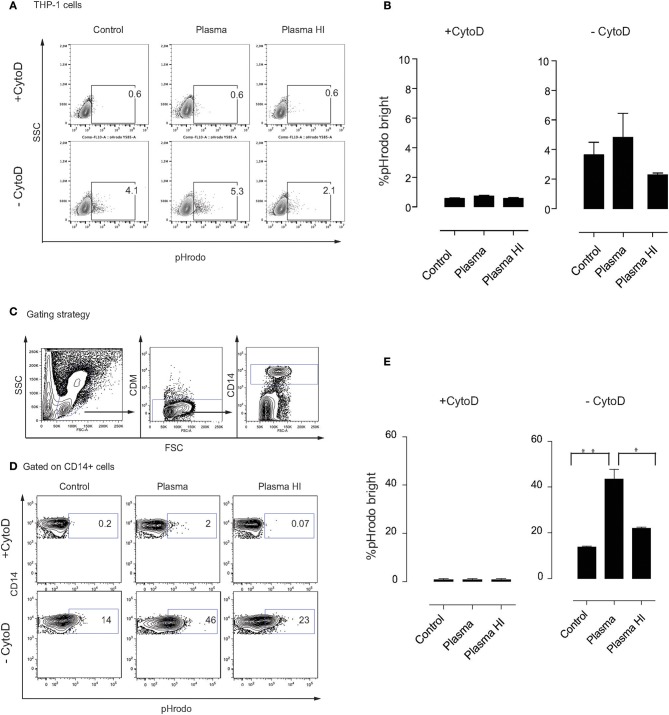
Complement enhances phagocytosis of MSC by monocytes. MSC were labeled with pHrodo and incubated with or without active plasma for 1 h, washed and co-cultured with THP-1 cells **(A,B)** or PBMC **(D,E)**. Phagocytosis was analyzed after 2 h co-culture. Phagocytosis inhibitor Cytochalasin D (CytoD) was added as a negative control. **(A)** Representative plots of flow-cytometric analysis of phagocytosis by THP-1 cells. pHrodo bright fluorescence indicates cells that have phagocytosed labeled MSC. **(B)** Pooled data of THP-1 cell phagocytosis, bars represent means with SD (*n* = 4) from two independent experiments. **(C)** Gating strategy of CD14^+^ monocytes in freshly isolated PBMC. **(D)** Representative plots of flow-cytometry analysis of phagocytosis by monocytes. pHrodo bright fluorescence indicates cells that have phagocytosed labeled MSC. **(E)** Pooled data from MSC phagocytosis by monocytes, bars represent means with SD (*n* = 4) of two independent experiments. Statistical significance was determined using ANOVA followed by Holm-Sidak's multiple comparisons test *p = 0.05, **p = 0.001.

Phagocytosis is considered to be an important pathway for removal of complement-opsonized cells from circulation (28). We hypothesized that MSC are phagocytosed via a mechanism that involves complement proteins. To address this, we labeled MSC with the pH-sensitive dye pHrodo, which has a low fluorescence at neutral pH that increases with decreasing pH, for instance upon entering phagolysosomes where the pH is significantly reduced (29). This approach allows to clearly address active phagocytosis of MSC by monocytes. As a negative control, Cytochalasin D (CytoD) was added to the co-culture in order to inhibit phagocytosis. We initially used the monocytic cell line THP-1, which has been extensively used in phagocytosis studies (30). Although the phagocytic capacity of THP-1 cells was not high, we consistently observed higher phagocytosis if MSC were pre-treated with plasma, compared to heat inactivated plasma or medium alone (Figures 3A,B). Therefore, we set-up experiments using fresh PBMC from healthy donors (Figures 3C–E) and gated on CD14^+^ cells in order to analyze phagocytosis by monocytes (Figure 3C). After 2 h of co-culture, around 15% of monocytes showed a higher pHrodo fluorescence indicating that MSC are phagocytosed by monocytes in the absence of plasma (Figures 3D,E). Interestingly, almost half of the monocytes phagocytosed MSC if they were pre-incubated with plasma and washed prior to co-culture (Figures 3D,E). When MSC were pre-incubated with HI plasma, we observed a significant decrease in phagocytosis of MSC by monocytes compared with plasma (Figures 3D,E). These results suggest that plasma factors interacting with MSC are responsible for the increased phagocytic capacity of monocytes.

### Phagocytosis of MSC Is Mediated by Classical and Intermediate Monocytes

**Figure 4:**
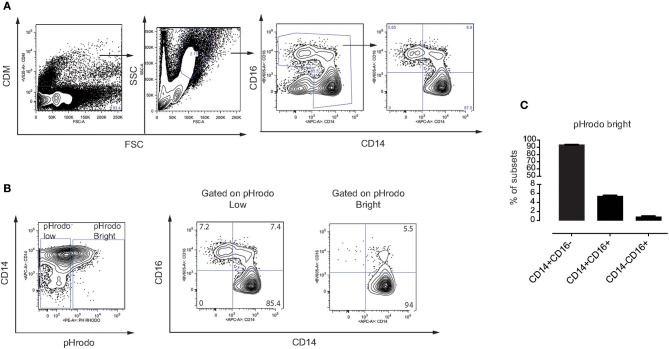
Phagocytosis of MSC is mediated by classical and intermediate monocytes. **(A)** Gating strategy on different subsets of monocytes in freshly isolated PBMC. Classical monocytes (CD14^+^ CD16^−^), intermediate monocytes (CD14^+^ CD16^+^), and non-classical monocytes (CD14^−^ CD16^+^). **(B)** Representative flow cytometry plots showing distribution of different subsets of monocytes on gated pHrodo Bright and pHrodo Low. **(C)** Distribution of the three monocyte subsets within the pHrodo Bright population. Bars represent mean values with SD (*n* = 4 PBMC) of pooled data from two different experiments.

In humans, monocytes can be divided into three subsets based on the expression of CD14 and the Fcγ receptor III CD16 (31), including classical monocytes (CD14^+^ CD16^−^), intermediate monocytes (CD14^+^ CD16^+^) and non-classical monocytes (CD14^−^ CD16^+^). Here, we made use of the CD14 and CD16 surface markers to identify these three subsets in our assays (Figure 4A). We addressed whether MSC phagocytosis, mediated by plasma factors, involves a specific subset of monocytes. Indeed, monocytes with phagocytosed MSC (pHrodo Bright) were mainly classical CD14^+^ CD16^−^ and intermediate CD14^+^ CD16^+^ monocytes while non-classical CD14^−^ CD16^+^ monocytes did not phagocytose MSC (Figures 4B,C) and Figure S3. Since intermediate monocytes represent only a small fraction of the total number of monocytes, the majority of pHrodo bright cells were classical monocytes (Figures 4B,C). The distribution of monocyte subsets that phagocytose MSC pre-treated with plasma was similar when we compared them to non-treated MSC or MSC treated with HI plasma (data not shown).

### Mechanism of MSC Phagocytosis by Monocytes

**Figure 5:**
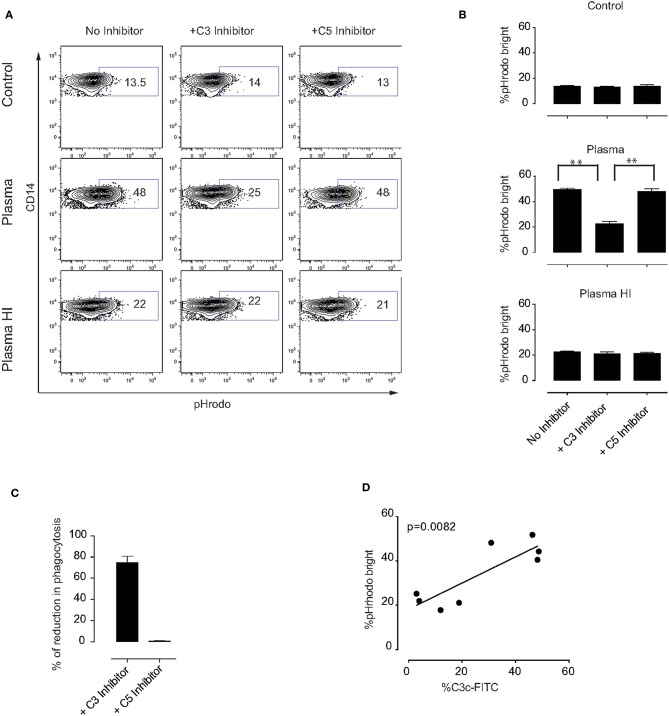
Mechanism of MSC phagocytosis by monocytes. MSC were labeled with pHrodo and incubated with or without active plasma for 1 h in the presence or absence of the C3 inhibitor compstatin or the C5 inhibitor soliris. Cells were washed and co-cultured with PBMC for 2 h. **(A)** Representative plots of flow-cytometric analysis of phagocytosis by monocytes. pHrodo bright fluorescence indicates cells that have phagocytosed labeled MSC. **(B)** Pooled data from two independent experiments using a total of four different MSC. Bars represent mean values with SD (*n* = 4). Statistical significance was determined using ANOVA followed by Holm-Sidak's multiple comparisons test **p = 0.001. **(C)** Percentage of reduction in phagocytosis in the presence of C3 or C5 inhibitor calculated from the data shown in **(B)** and the percentage of phagocytosis observed in plasma free medium was subtracted. **(D)** Correlation between percentage of C3b/iC3b binding to MSC and percentage of pHrodo bright monocytes from HI plasma and plasma in the phagocytosis experiments.

**Figure 6:**
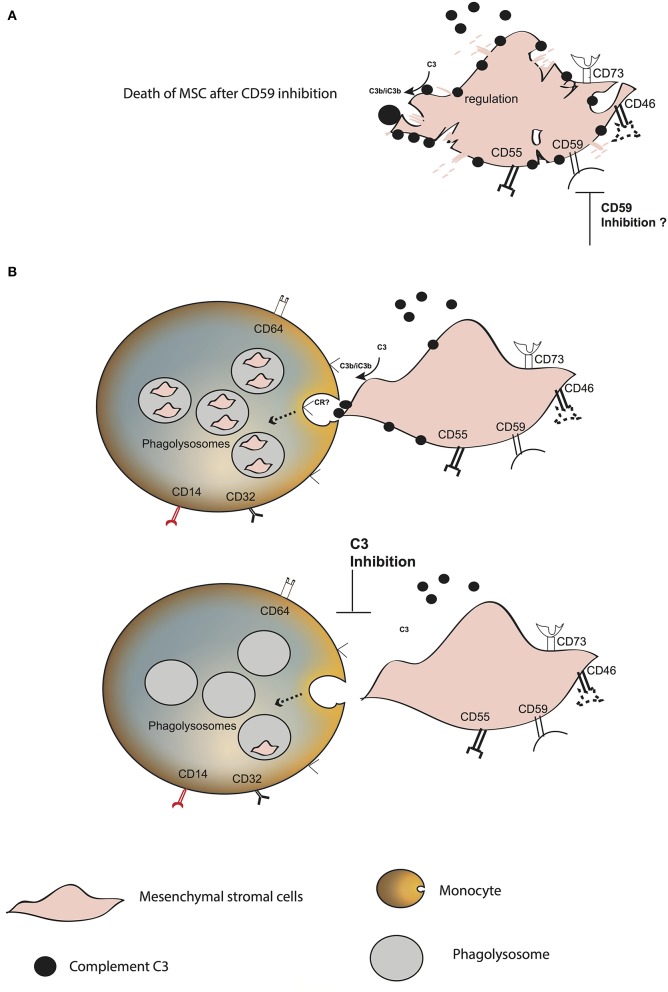
Model of MSC fate after interaction with blood. **(A)** Increase in C3 binding and decrease in the survival of plasma pre-treated MSC in the presence of anti-CD59. **(B)** C3 binds to the surface of MSC, probably through the alternative complement pathway. CD59 blocks the membrane attack complex from forming. MSC are phagocytosed by classical and intermediate monocytes, mainly mediated by the presence of C3 on the MSC surface. The receptor binding to C3 and inducing the phagocytosis remains unknown.

To better understand which plasma components are involved in the phagocytosis by monocytes of MSC pre-treated with plasma, we first tested whether complement factors may play a role in this process. The complement factors C3 and C5 were selected due to their important roles in monocyte-mediated phagocytosis (17, 20, 32). As anticipated, inhibition of complement factors C3 or C5 did not change the percentage of phagocytosis of control MSC or MSC pre-incubated with HI plasma (Figures 5A,B). However, we observed a significant decrease in the phagocytosis of MSC pre-incubated with plasma in the presence of a C3 inhibitor. On the other hand, inhibition of C5 had no effect on monocyte phagocytosis (Figures 5A,B). Following subtraction of basal phagocytosis of MSC (phagocytosis which is observed in plasma free medium), ~80% of plasma-mediated increase of phagocytosis was due to C3 binding (Figure 5C). In addition, a significant positive correlation between C3b/iC3b binding and the percentage of pHrodo bright monocytes was detected (Figure 5D). Altogether, these results indicate that C3 is an important mediator for phagocytosis of MSC by monocytes (Figure 6). Further investigations are required to unravel the exact molecular mechanisms underlying the role of C3 in phagocytosis of MSC by monocytes.

## Discussion

The current consensus in the field of MSC therapy has hitherto been that intravenous infusion of MSC leads to quick clearance of MSC from the blood circulation. The remaining debate has been focused on the fate of administrated MSC (5). Here, we report that MSC survive and conserve their phenotypic and functional activities if they are in contact with complement active plasma. Furthermore, our results demonstrate that the complement factor C3 facilitates phagocytosis of live MSC by classical and intermediate monocytes.

One of the first lines of innate defense of the immune system is the complement system, which during infection or entry of foreign cells will bind to their cell surface and amplify a cascade of enzymatic reactions (16). To prevent uncontrolled complement activation, cells express a number of complement regulators at their surface (18). Based on our and other previous reports showing that MSC express complement inhibitors including CD46, CD55, and CD59 (14, 15, 33), we hypothesized that MSC are protected from complement attacks. Our results suggest that CD59 might protect MSC from complement lysis. However, this result must be taken with caution since the specific isotype (IgG2b) of the anti-CD59 antibody used within the present study may lead to the activation of the classical complement pathway as previously reported (27).

On the other hand, our results are also in accordance with Feng et al. who demonstrated that CD59 plays an important role in protection against complement-mediated cytotoxicity (15). However, the same report also suggested a role for CD55, which we did not observe in our study. The latter could be due to different expression levels of CD46, CD55, and CD59 on the surface of MSC. Furthermore, it has also been shown that despite CD59 and CD55 expression, MSC were injured after complement binding, as revealed by release of the fluorescent dye bis-carboxyethyl-carboxyfluorescein (BCECF) from MSC (15). In our study, we did not observe calcein leakage when MSC were treated with plasma suggesting that MSC were not injured. Thus, it is plausible that the different dyes used to detect cytotoxicity might be the reason for the different results obtained. Indeed, calcein has been reported to display higher sensitivity and less spontaneous leakage than BCECF (34). Moreover, plasma pre-treated MSC stained negative for all the three commonly used cell death markers in both flow cytometry and FlowSight techniques and displayed full functional capability favoring the hypothesis that MSC survive after contact with plasma. Our data are in accordance with mouse and human *in vivo* tracing experiments, which identified MSC in different organs hours to days after infusion (7, 8, 21, 35).

We observed an increase in C3b/iC3b deposition on MSC after CD59 inhibition. Here again, we are not excluding the clear possibility that this could be due to the activation of the classical complement pathway (27). However, recent findings suggest a role for CD59 not only as a major controller of the membrane attack complex (19) but also in C3 regulation (36, 37). Furthermore, although CD55 was suggested to regulate C3 (36), we did not observe any significant changes in C3b/iC3b deposition when CD55 was blocked on MSC. More experiments are therefore required for further clarification. It should be noted that interaction with complement is not unique to bone marrow MSC as adipocyte stromal cells have been shown to interact with complement in rat peritonitis model (38).

Complement deposition on the cell surface serves as target for complement receptors present on mononuclear phagocytic cells in particular monocytes and macrophages (28). Our results revealed that live MSC were targeted by monocytes via a mechanism that involves complement. Such a mechanism might explain in part the observed rapid clearance of MSC when infused to the circulation. Indeed, only 2 h of incubation led to phagocytosis of complement-opsonized MSC by more than 45% of monocytes. A recent study by the Hoogduijn research group used umbilical cord MSC (uMSC) labeled with the lipophilic membrane dye PKH26, which were mixed with monocytes in blood. They found PKH26-labeled uMSC fragments on monocytes 3 h after co-culture (22). Also, Braza et al. found in an asthma model in mice that injected PKH26-labeled MSC were engulfed by lung macrophages within 24 h following *i.v*. injection (21). However, the use of PKH26 may be disadvantageous since it also could incorporate itself into other cells (in this case to monocytes or macrophages) through a cell-to-cell membrane transfer process called trogocytosis, giving rise to false positive signals without phagocytosis (39, 40). In the current study, MSC were labeled with pHrodo, which has the advantage that it is only fluorescent under acidic conditions, consequently it will only be fluorescent in phagolysosomes (pH 4) but neither outside of the cell nor in the cytoplasm where the pH is around 7 (29, 41). Thus, our data demonstrated that live complement-opsonized bone marrow MSC were indeed phagocytosed by monocytes. Intriguingly, we showed that MSC were also phagocytosed by monocytes in the absence of plasma albeit to a lesser extent. These observations are consistent with the paradigm that MSC can be engulfed by cancer cells in the absence of plasma (42). One possible explanation might be that MSC express adhesion molecules that allow a tight contact with monocytes, which could facilitate their phagocytosis (28, 43). A recent study suggested that apoptosis of MSC is induced by cytotoxic T cells which favor their engulfment by phagocytic cells (13). Our data complement this study and reveal that live MSC can also be subjected to phagocytosis by monocytes. This might explain recent pre-clinical studies showing positive effect of living MSC in the treatment of sepsis (44, 45). Nevertheless, a comparative study on the immunomodulatory potential of monocytes which phagocyte apoptotic vs. live MSC is required.

With further evaluation of the mechanism of complement-mediated phagocytosis, we found that addition of compstatin, which blocks the activation of complement at the C3 level (32) significantly affected phagocytosis of MSC by monocytes. Adding the C3 inhibitor reduced C3b/iC3b deposition on the surface of MSC suggesting that binding of C3 at the surface of MSC is associated with complement activation confirming previous observations (14). However, the exact mechanisms underlying how C3 deposition triggers phagocytosis remain to be investigated. An interesting question, which needs further investigation, is which receptor on monocytes is involved in MSC phagocytosis. Monocytes express receptors for different C3 derivative fragments including CR1 (CD35), CR2 (CD21), CR3 (CD11b/CD18), and CR4 (CD11c/CD18) (46). Among these receptors, CR1 and CR2 on monocytes are involved in phagocytosis via interactions with the C3 complement during infection (47). Thus, a similar mechanism might occur for MSC. Interestingly, phagocytosis mediated via complement receptors is not always associated with inflammation (47). Therefore, it is reasonable to speculate that monocytes might be the final destination of MSC after their infusion into the circulation without inducing an excessive inflammation. This might partly explain the mild inflammation detected after intravenous infusion of MSC (48).

Another question addressed in our study is which type of monocytes phagocyte MSC. Moll et al. showed that depletion of CD14^+^CD11b^high^ monocytes was associated with strong decrease in the immunosuppressive function of MSC *in vitro* in both alloantigen- and PHA-stimulated mixed lymphocyte reactions (14). We found that classical CD16^−^ CD14^+^ and intermediate CD16^+^ CD14^+^ monocytes were first in engulfing MSC supporting recent findings (22). However, we did not see any significant changes in expression of the other Fc receptors CD32 or CD64 on phagocytic monocytes which have been involved in complement-mediated phagocytosis during certain infection (data not shown). However, kinetic experiments are needed to see whether non-classical CD16^+^ CD14^−^ monocytes are also involved in the phagocytosis of MSC at a later time point. Indeed, de Witte et al. suggested that 24 h after engulfment of uMSC, monocytes polarize from CD14^+^ CD16^−^ to CD14^+^ CD16^+^ expressing cells (22).

In conclusion, we propose that complement opsonization plays a crucial role in the fate of MSC after intravenous infusion (Figure 6). It mediates their rapid phagocytosis by classical and intermediate CD14^+^ monocytes. MSC are protected against complement injury through CD59, which is in contrast to the previous dogma that MSC disappear from circulation due to destruction. Our results provide new insights on the fate of MSC after intravenous infusion, which needs to be taken into consideration in order to improve the therapeutic role of MSC in various diseases.

## Data Availability Statement

All datasets generated for this study are included in the manuscript/Supplementary Files.

## Ethics Statement

The studies involving human participants were reviewed and approved by Ethics committee at Karolinska Institutet. The patients/participants provided their written informed consent to participate in this study. The following are the three ethical permits that cover whole paper. DNR: 2013/39-31/4, DNR: 446/00, and DNR: 2016/338-32-4.

## Author Contributions

This study was designed by CG, KL, and NK with the input of SM, NH, EI, AA, PH, and KH. CG, EI, KH, SM, and NK performed experiments, analyzed, and interpreted the results. CG, SM, KL, and NK wrote the manuscript with inputs from EI, PH, and AA. All authors read, commented, and approved the final manuscript.

### Conflict of Interest

The authors declare that the research was conducted in the absence of any commercial or financial relationships that could be construed as a potential conflict of interest.

## Supplementary Material

Figure S1 Complement inhibitors CD46, CD55, or CD59 are not toxic on MSC. Representative contour plots of calcein RO stained MSC and exposed to plasma, HI plasma or medium for 1 h in the presence or absence of complement inhibitors CD46, CD55, or CD59. Data is representative of 5 MSC of two independent experiments.

Figure S2 Representative contour plots showing shape (FSS) and granulosity (SSC) of MSC after incubation for one hour with or without active plasma, in the presence or absence of complement inhibitor anti-CD59. Data are representative of at least three different experiments.

Figure S3 Phagocytosis of MSC is mediated by classical and intermediate monocytes. Presence of non classical monocytes among other subsets on gated pHrodo Low MSC. Pooled data of two different donors (PBMC = 4).
