# Supplementary figures and images for: The Complement System Is Essential for the Phagocytosis of Mesenchymal Stromal Cells by Monocytes
Source: Front Immunol. 2019 Sep 20;10:2249. doi: 10.3389/fimmu.2019.02249 (PMC6763726; doi:10.3389/fimmu.2019.02249)

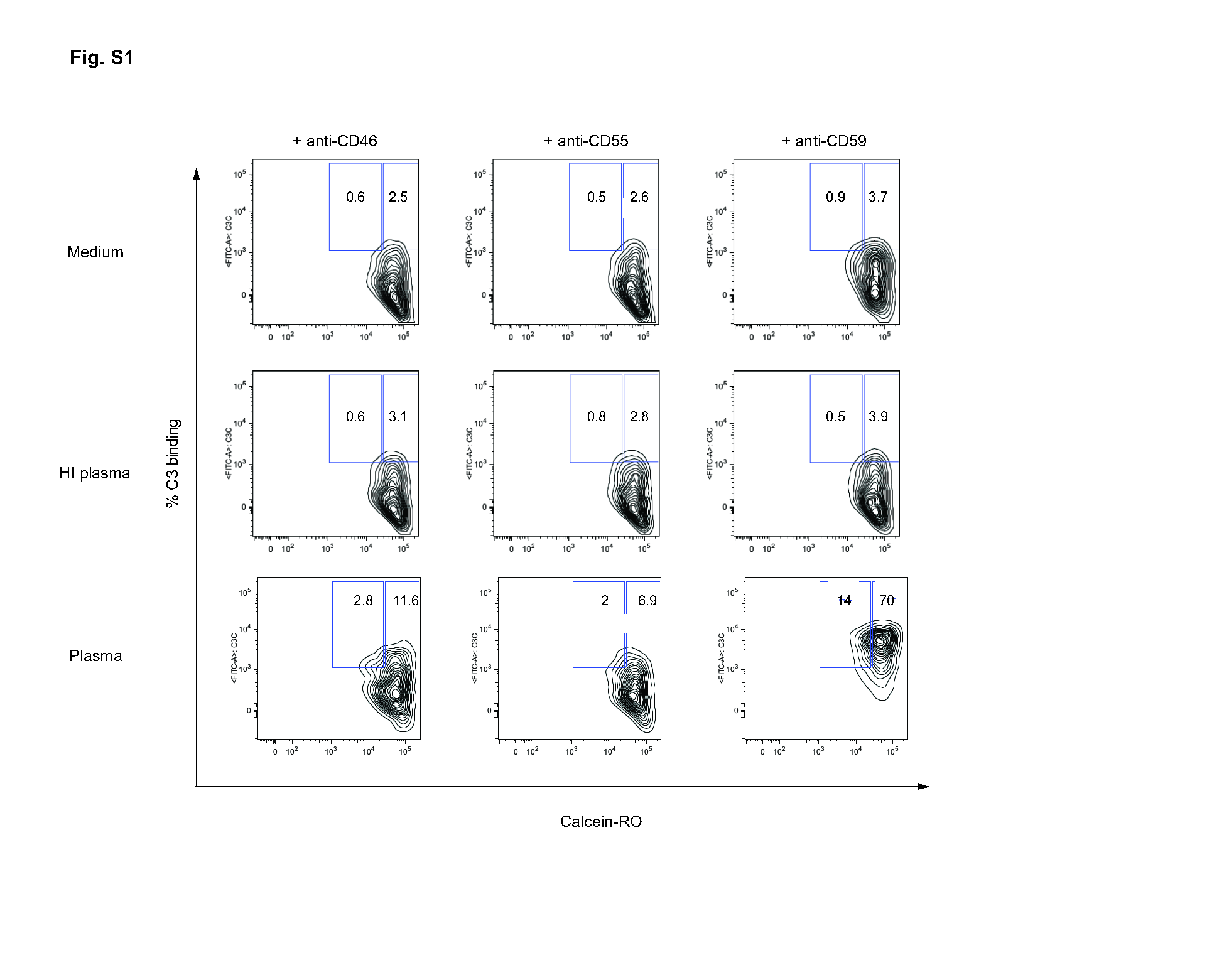

Supplement: Figure S1 — Complement inhibitors CD46, CD55, or CD59 are not toxic on MSC. Representative contour plots of calcein RO stained MSC and exposed to plasma, HI plasma or medium for 1 h in the presence or absence of complement inhibitors CD46, CD55, or CD59. Data is representative of 5 MSC of two independent experiments. [file Image_1.TIFF]

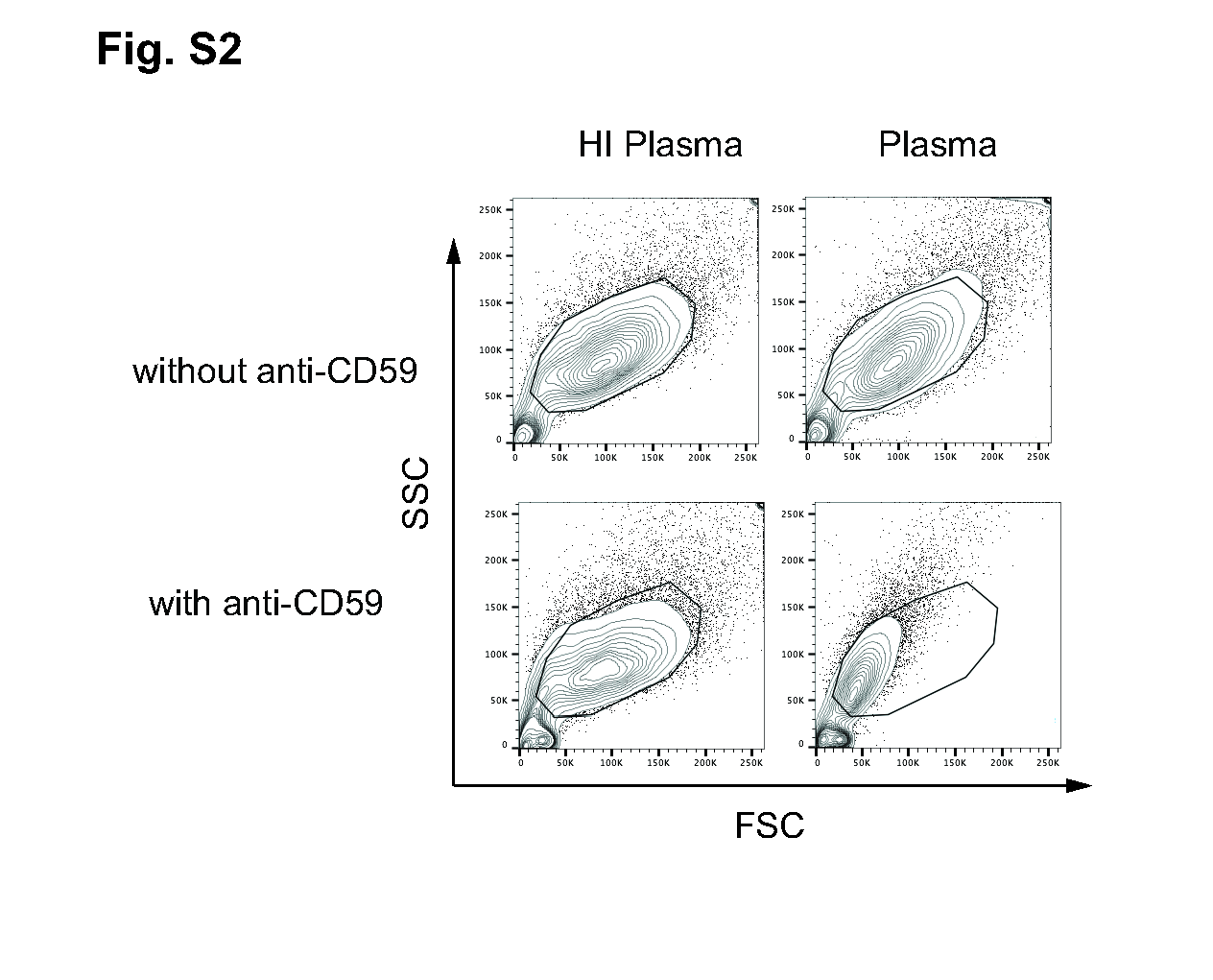

Supplement: Figure S2 — Representative contour plots showing shape (FSS) and granulosity (SSC) of MSC after incubation for one hour with or without active plasma, in the presence or absence of complement inhibitor anti-CD59. Data are representative of at least three different experiments. [file Image_2.TIFF]

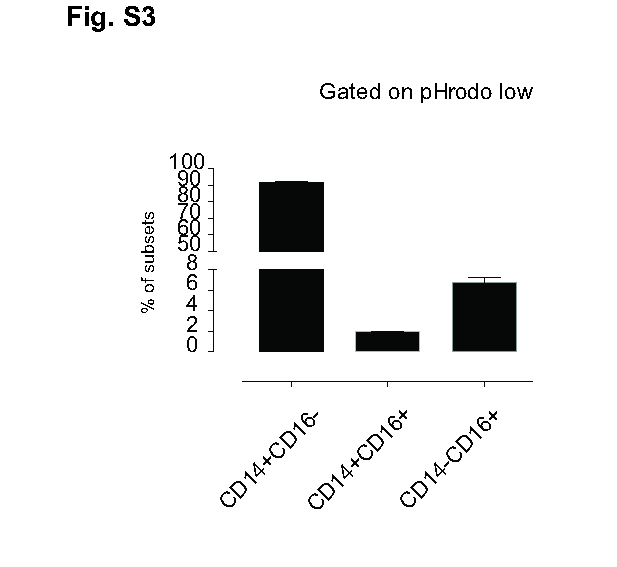

Supplement: Figure S3 — Phagocytosis of MSC is mediated by classical and intermediate monocytes. Presence of non classical monocytes among other subsets on gated pHrodo Low MSC. Pooled data of two different donors (PBMC = 4). [file Image_3.TIFF]
